# Pathological ATPergic Signaling in Major Depression and Bipolar Disorder

**DOI:** 10.3389/fnmol.2019.00331

**Published:** 2020-01-31

**Authors:** Peter Illes, Alexei Verkhratsky, Yong Tang

**Affiliations:** ^1^Rudolf-Boehm-Institut für Pharmakologie und Toxikologie, Universität Leipzig, Leipzig, Germany; ^2^Acupuncture and Tuina School, Chengdu University of Traditional Chinese Medicine, Chengdu, China; ^3^Faculty of Life Sciences, The University of Manchester, Manchester, United Kingdom; ^4^Achucarro Centre for Neuroscience, Ikerbasque, Basque Foundation for Science, Bilbao, Spain

**Keywords:** P2X7 receptor, mood disorders, hippocampus, microglia, astroglia

## Abstract

The mood disorders, major depression (MD) and bipolar disorder (BD), have a high lifetime prevalence in the human population and accordingly generate huge costs for health care. Efficient, rapidly acting, and side-effect-free pharmaceuticals are hitherto not available, and therefore, the identification of new therapeutic targets is an imperative task for (pre)clinical research. Such a target may be the purinergic P2X7 receptor (P2X7R), which is localized in the central nervous system (CNS) at microglial and neuroglial cells mediating neuroinflammation. MD and BD are due to neuroinflammation caused in the first line by the release of the pro-inflammatory cytokine interleukin-1β (IL-1β) from the microglia. IL-1β in turn induces the secretion of corticotropin-releasing hormone (CRH) and in consequence the secretion of adrenocorticotropic hormone (ACTH) and cortisol, which together with a plethora of further cytokines/chemokines lead to mood disorders. A number of biochemical/molecular biological measurements including the use of P2X7R- or IL-1β-deficient mice confirmed this chain of events. More recent studies showed that a decrease in the astrocytic release of ATP in the prefrontal cortex and hippocampus is a major cause of mood disorders. It is an attractive hypothesis that compensatory increases in P2X7Rs in these areas of the brain are the immediate actuators of MD and BD. Hence, blood-brain barrier-permeable P2X7R antagonists may be promising therapeutic tools to improve depressive disorders in humans.

## Introduction

The mood disorder major depression (MD) is characterized by extreme sadness, depressed mood, and loss of interest that persist for at least 2 weeks and interferes with the individual’s social functioning (Harvey et al., 2007; Deussing and Arzt, 2018; Wei et al., 2018; Ribeiro et al., 2019). During bipolar disorder (BD), the mood state cycles between high (mania) and low (depression) episodes. MD and BD arise from complex interactions between genetic, developmental, and environmental factors (Koenig et al., 2011; Sullivan et al., 2012). The lifetime prevalence estimates for MD vary from 11% to 14% with females having an approximately 2-fold higher disease risk than males (Deussing and Arzt, 2018).

In view of the serious limitations these mood disorders impose upon the life quality of patients and because of their relatively frequent occurrence in the human population, it is of eminent importance to find good curative strategies to combat them. Presently, reuptake inhibitors of monoamines [noradrenaline, dopamine, and 5-hydroxytryptamine (5-HT)] are in the forefront of considerations, although significant drawbacks have to be taken into account: (1) the clinical improvement is achieved only after weeks of treatment; (2) there are multiple side effects; and (3) a substantial group of patients is resistant to therapy (Kulkarni and Dhir, 2009; Deussing and Arzt, 2018). Therefore, intensive search for alternative therapeutic targets and tools is a compelling necessity.

## Purinergic P2X7 Receptor

Ionotropic P2X7 receptors (P2X7Rs) are members of the P2X purinoceptor family, which were cloned and characterized in 1996 (Surprenant et al., 1996; North, 2002; Burnstock and Knight, 2004). Three properties of the P2X7R are distinguishing characteristics: (1) it is activated by high concentrations of ATP in the millimolar range, clearly surmounting concentrations needed to activate other P2X receptors (P2XRs), which are stimulated by ATP concentrations in the micromolar range; (2) it is a ligand-gated cationic channel, allowing the inward passage of Na^+^ and Ca^2+^ and the outward passage of K^+^ through the cell membrane. However, its repetitive or longer-lasting activation by ATP results in the opening of membrane pores permeable to large organic cations such as the fluorescent dye YO-PRO, which otherwise do not pass the cell membrane; and (3) the P2X7R consists of three subunits (large extracellular loop, two transmembrane regions, and N- and C-terminal ends) forming a receptor, but each subunit has a much longer C-terminus than that of the other P2XRs.

A particularly intensively discussed issue is the transition of the cationic channel to a large membrane pore, because it appears to be essential for cytokine production and secretion (Illes et al., 2019; Martin et al., 2019). Originally, it was suggested based on equilibrium potential (*V*_rev_) measurements with the whole-cell patch-clamp technique that the ion conducting pathway shows progressive dilation (Virginio et al., 1999). However, this suggestion was recently refuted, because the shift in *V*_rev_ in a medium in which the counterion of intracellular K^+^ was NMDG^+^ instead of Na^+^, emerged due to time-dependent alterations in the concentration of intracellular ions rather than channel dilation (Li et al., 2015). Moreover, during long-lasting activation of P2X7Rs, the single-channel current amplitude and the permeation characteristics remained constant (Pippel et al., 2017). Although convincing evidence indicates that pore opening is due to the recruitment of an accessory protein, the pannexin-1 channel (Panx-1; Pelegrin and Surprenant, 2006; Gulbransen et al., 2012; Shoji et al., 2014; Chen et al., 2017), the observation that, for example, the P2X7R pore formation is retained in Panx-1^−/−^ cells supports the opposite notion (Hanley et al., 2012).

The P2X7R C-terminal tail constitutes about 40% of the whole protein, and its deletion or massive truncation prevents effects mediated by receptor activation such as dye uptake and membrane blebbing (generation of exosomes) but also alters channel kinetics (Kopp et al., 2019). In addition, the C-terminus was implicated in regulating signaling pathway activation, protein–protein interactions, and posttranslational modification (Costa-Junior et al., 2011).

**Figure 1:**
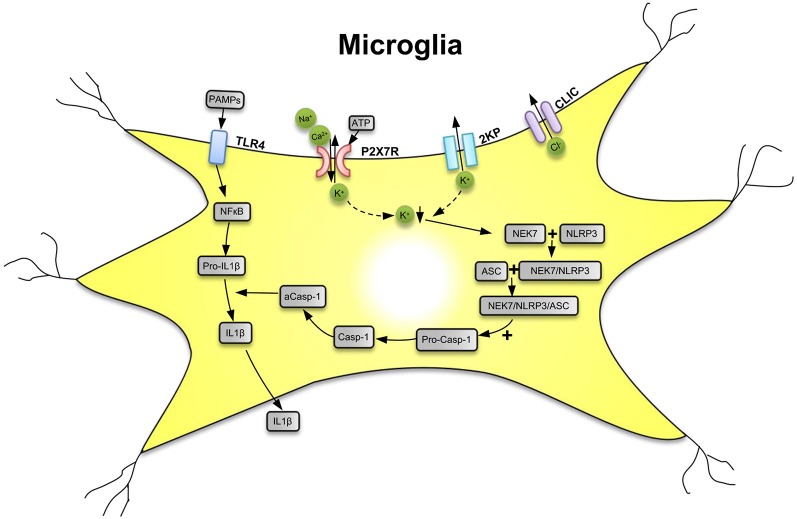
Secretion of interleukin-1β (IL-1β) from microglial cells *via* involvement of the nucleotide-binding, leucine-rich repeat, pyrin domain containing 3 (NLRP3) inflammasome. Pathogen-associated molecular patterns [PAMPs; e.g., bacterial lipopolysaccharide (LPS)] act on toll-like receptor-4 (TLR4) and cause its phosphorylation. In consequence, in the cell nucleus, NF-κB is activated, which promotes the synthesis of the NLRP3 inflammasome and pro-IL-1β, both accumulating in the cytosol in their inactive forms. The activation of NLRP3 is primarily due to a decrease of the intracellular K^+^ concentration ([K^+^]_i_), initiated by the stimulation of P2X7Rs by high local concentrations of the molecule ATP, which is considered to be a danger-associated molecular pattern (DAMP). P2X7Rs allow the inward flux of Na^+^/Ca^2+^ and in exchange the outward flux of K^+^, leading to a fall in [K^+^]_i_. The opening of two-pore domain potassium channels (2KP) may also lead to an impoverishment in cytoplasmic K^+^. A further stimulus for NLRP3 activation is the outward flux of Cl^−^ through chloride intracellular channels (CLICs). TLR4, P2X7Rs, 2KP channels, and CLIC are all located in the cell membrane of the microglia. A sensor for the fall in [K^+^]_i_ is the NEC7 serine/threonine kinase. NEC7 is able to form a complex with NLRP3, which is still inactive, but after constitution of a still larger multimeric complex with apoptosis-associated speck-like protein (ASC) recruits pro-caspase-1 (pro-Casp-1). In consequence, pro-Casp-1 in a complex with NLRP3 and ASC is cleaved to Casp-1, which then by its activated form a-Casp-1 degrades pro-IL-1β to IL-1β. Then, IL-1β leaves the cell by a number of mechanisms to the extracellular space and exerts its effects as a neuroinflammatory cytokine. K^+^ ↓, decrease of the K^+^ concentration. Artwork by Dr. Hayan Yin.

P2X7Rs are major drivers of inflammation (Di Virgilio et al., 2017; Burnstock and Knight, 2018; Savio et al., 2018). Secretion of several pro-inflammatory cytokines and chemokines depends on the activation of P2X7Rs by large concentrations of ATP outpouring from damaged central nervous system (CNS) cells. The preferential location of P2X7Rs in the CNS is on the microglia, the resident macrophages of the brain (Bhattacharya and Jones, 2018). Microglia are equipped with a battery of pattern recognition receptors that stereotypically detect pathogen-associated molecules (PAMPs) such as lipopolysaccharide (LPS) from bacterial infection or danger-associated molecular patterns (DAMPs), such as ATP (Figure 1; Shao et al., 2015; Young and Górecki, 2018; Illes et al., 2019; Martin et al., 2019). Activation of microglia stimulates the release of interleukin-1β (IL-1β) in a two-step process: the first being the stimulation of toll-like receptor 4 (TLR4) by LPS, leading to accumulation of cytoplasmic pro-IL-1β, and the second being the ATP-dependent stimulation of P2X7Rs, promoting nucleotide-binding, leucine-rich repeat, pyrin domain containing 3 (NLRP3) inflammasome-mediated caspase-1 activation and secretion of IL-1β (Perregaux and Gabel, 1998; Ferrari et al., 2006). Caspase-1 generates IL-1β from pro-IL-1β by enzymatic degradation. It is important to note that the decrease of intracellular K^+^ is a major stimulus for P2X7R-dependent NLRP3 inflammasome activation (Muñoz-Planillo et al., 2013; Di Virgilio et al., 2017, 2018).

IL-1β is co-produced/secreted with other pro-inflammatory cytokines such as IL-6 and IL-18 as well as tumor necrosis factor-α (TNF-α). A convincing argument for the idea that P2X7R activation provides the signal that leads to maturation and release of IL-1β and initiation of the cytokine cascade stemmed from experiments showing that P2X7R^−/−^ cells or animals primed with LPS failed to produce IL-1β on the application/injection of ATP (Solle et al., 2001).

The majority of the fully sequenced mammalian genomes include representatives of all vertebrate P2X genes, including P2X4, which in humans is located on chromosome 12 in close proximity to P2X7 (Suurväli et al., 2017). The overlapping expression of P2X4 and P2X7Rs has been documented in macrophages and microglia (Dubyak, 2007; Suurväli et al., 2017). The reason for the co-expression may be the involvement of both receptors in inflammatory processes (de Rivero Vaccari et al., 2012; Hung et al., 2013; Sakaki et al., 2013). Originally, it has been assumed that subunits of P2X4 and P2X7Rs form the heteromeric complex P2X4/P2X7 (Guo et al., 2007), although more recent data lend support to the existence of independent receptors tightly interacting with each other (Nicke, 2008; Antonio et al., 2011). The agonist binding affinities largely differ between P2X7 and P2X4 receptors (P2X4Rs); while the former one is activated by millimolar ATP concentrations, the latter one responds to ATP in the micromolar range (Kaczmarek-Hájek et al., 2012). Hence, non-cell-lytic micromolar ATP release cannot directly stimulate P2X7Rs but easily activates its more sensitive partner, the P2X4R, thereby modifying the function of the P2X4–P2X7R multiprotein complex.

## Association of P2X7 Gene Polymorphism and Mood Disorders

Linkage studies suggested that variations of the chromosome 12q24,31 containing candidate genes for the P2X7R and calmodulin-dependent protein kinase b (CaMKKb) may be associated with MD and BD. It has been repeatedly reported that the nonsynonymous single-nucleotide polymorphism (NS-SNP) *rs2230912* coding for Gln460Arg-P2X7R is associated with MD (McQuillin et al., 2009; Soronen et al., 2011; Sperlágh and Illes, 2014). However, in the meantime this association has been questioned. Although further studies have supported the possible role of this NS-SNP in mood disorders (Halmai et al., 2013; Vereczkei et al., 2019), other authors failed to detect any association (Green et al., 2009; Grigoroiu-Serbanescu et al., 2009). Two recent meta-analyses also yielded divergent results, one of them confirming (Czamara et al., 2018) and the other one refuting (Feng et al., 2014) the hypothesis on the causal role of the NS-SNP *rs2230912* in MD and BD. Eventually, this led the Psychiatric Genomics Consortium to deny the *P2RX7* gene as a genetic risk factor for mood disorders in large-scale genome-wide association studies (Mühleisen et al., 2014; Wray et al., 2018).

When various *P2RX7* single-nucleotide polymorphism were investigated by electrophysiology/dye uptake studies either in native cells or in HEK293 cells transfected with the respective plasmids, several gain-of-function or loss-of-function allelic mutations were identified (Gu et al., 2001; Roger et al., 2010; Sun et al., 2010). Surprisingly, the ATP-induced inward current was the same through the wild-type (WT) receptor and the Gln460Arg polymorphic receptor (Roger et al., 2010), leading to the suggestion that a haplotype block may explain the lack of the expected decrease of ATP effects (Sluyter et al., 2010). This riddle was dissolved by Aprile-Garcia et al. (2016) who reported that although the variant *per se* is not compromised in its function, co-expression of WT P2X7R with the Gln460Arg-P2X7R results in inhibition of calcium influx, channel current, and intracellular signaling. Moreover, co-immunoprecipitation and FRET studies demonstrated that the Gln460Arg-P2X7R variant physically interacts with the WT P2X7R. The same group of authors found that humanized mice co-expressing both P2X7R variants showed alterations in their sleep quality resembling signs of a prodromal MD state (Metzger et al., 2017a).

In conclusion, the evidence for an association of the SNP *rs2230912* as an etiologic factor for hereditary mood disorders is far from being equivocal, although its role in P2X7R involvement cannot be excluded either (see above controversial results of epidemiological studies).

## The P2X7R Triggers Neuroinflammation and Subsequent Mood Disorders

Activation of the inflammasome, which precipitates the release of pro-inflammatory cytokines, and activation and migration of microglia and reactive astrogliosis are key regulators of the neuroinflammatory response (Beamer et al., 2016; Liu and Quan, 2018). IL-1β is a master regulator of inflammatory reactions, capable of activating innate immunity by inducing the expression of inflammatory cytokines and chemokines, eliciting leukocyte infiltration into the inflammatory loci, increasing the phagocytic and bactericidal activity of immune cells, enhancing the activity of the complement system, and facilitating the activation of the adaptive immune responses (Dinarello, 2009; Liu and Quan, 2018).

Stress exposure is considered to be the main environmental factor instigating mood disorders in humans, and all animal models of MD are based on exposure to inescapable stress (Ribeiro et al., 2019). Psychological and metabolic stress could induce adrenocorticotropic hormone (ACTH) and glucocorticoid secretion in mice, which were reduced in IL-1 knockouts (KOs) or transgenic animals overexpressing brain IL-1ra, a naturally occurring IL-1 antagonist (Goshen et al., 2003; Liu and Quan, 2018). Intracerebral administration of IL-1 induces corticotropin-releasing hormone (CRH) release in rats (Barbanel et al., 1990), and psychological stress causes brain IL-1 expression (Gadek-Michalska and Bugajski, 2010). Thus, brain IL-1 could mediate physiological responses to stress by stimulating the production of the immunosuppressive glucocorticoid hormone cortisol from the adrenal medulla (Liu and Quan, 2018). In perfect correlation with this idea, IL-1ra suppresses stress-induced depression in animal models (Koo and Duman, 2009; Maes et al., 2012). Consequently, disturbances of the main neuroendocrine stress response system, the hypothalamic–pituitary–adrenal axis including the main initiator CRH and effector glucocorticoids, have been suggested to cause depression (de Kloet et al., 2005; Deussing and Arzt, 2018).

As outlined previously, the primary function of P2X7Rs in the CNS is to initiate (neuro)inflammation. Therefore, it was deduced that the receptor might cause MD and BD, which are reportedly accompanied by neuroimmunological alterations (Bhattacharya and Jones, 2018). The chain of events may be the following: stress causes a massive outflow of ATP in the brain stimulating P2X7Rs, which on their behalf trigger the release of IL-1β. Then, IL-1β induces the secretion of CRH and the consecutive production of ACTH/glucocorticoids, resulting in mood disorders. In fact, acute restraint stress rapidly increases extracellular ATP, the inflammatory cytokine IL-1β, and the active form of the NLRP3 inflammasome in the hippocampus of rodents (Iwata et al., 2016).

**Figure 2:**
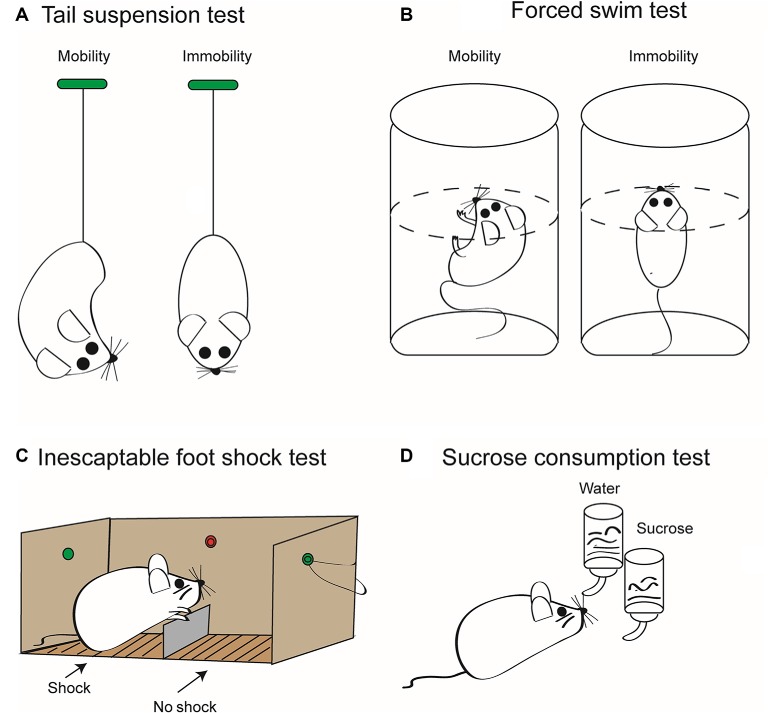
Some relevant tests to measure depressive-like behavior in rodents induced by stressors. These tests are employed to quantify the extent of “learned helplessness” of rats/mice, and thereby, with the necessary precaution, they are supposed to model major depression (MD) in humans. In consequence, they are routinely used to determine the effectiveness of antidepressant pharmacological agents. **(A)** Tail suspension test (TST). Mice are suspended by their tails with tape, in such a position that they cannot escape or hold on to nearby surfaces. Then, the sum of the time periods is measured during which they stop escape reactions; that is, they become immobile. This time period is considered to be a measure of the depressive-like behavior. The duration of the test is maximized usually at 6 min, in order to avoid unnecessary suffering of the animals. Because the weight of rats is much larger than that of mice, rats are not considered to be an appropriate rodent species for this test method. **(B)** Forced swim test (FST). Mice or rats are put into a tank containing water whose temperature is kept at about 23°C. The dimensions of the tank and the depth of water are such that the animals are forced to swim as an escape reaction. Swimming is stopped when the animal notices that it cannot escape and starts to float on the surface of the water. The length of the immobile periods is measured during a maximum of 6 min and is considered to be a measure of the degree of depressive-like behavior. **(C)** Inescapable foot shock test (IFST). The electric foot shock paradigm includes acute or chronic exposures of shocks of varying intensity and duration on an electrified grid floor in a foot shock apparatus. In contrast to the scheme shown, the mice or rat is not able to escape from the chamber where it is subjected to electric shocks to the other chamber where there is no comparable painful stimulation. Animals generally do not habituate to foot shocks in comparison to other stressors, including loud noise, bright light, and hot and cold temperatures. **(D)** Sucrose consumption test (SCT). The two-bottle choice procedure for assessing sucrose preference is a useful test to investigate anhedonia (i.e., inability to feel pleasure) in laboratory rodents. It allows for a comparison between the preference for sucrose solution in drinking water and that for water only. This preference is measured by volume and/or weight of liquid consumed daily, which is then converted to a percent sucrose solution consumed compared to a water only baseline period. As a result of the anhedonia induced by inescapable foot shock, the preference for choosing a sucrose solution decreases in mice or rats. It is important to verify the results of all these tests with separate behavioral tests that measure overall activity such as the open-field test. Moreover, TST, FST, and IFST/SCT should be used in combination to minimize false positivity, and it should be kept in mind that depressive-like behavior in laboratory rodents is not identical with the clinical state of MD in human beings (see above). Artwork by Ms. Lumei Huang.

Acute and chronic stress may induce in rodent models depressive-like behavior, which can be used to investigate antidepressive pharmaceuticals (Figure 2). In contrast to the acute stress models shown in this figure, unpredictable chronic mild stress (UCMS) is delivered for prolonged periods of 8–12 weeks and includes once daily, for example, immobilization, food deprivation, light/dark phase reversal, hot environment, and cage shaking. This procedure leads to depressive-like behavior as measured by reduced sucrose consumption and prolonged immobility in the tail suspension test (TST) and forced swim test (FST) in rodents (Zhang et al., 2015; Su et al., 2017; Wang et al., 2018; Feng et al., 2019). UCMS also resulted in higher protein levels of NLRP3, caspase-1, and IL-1β in the hippocampus of stress-exposed mice (Zhang et al., 2015) and rats (Wang et al., 2018; Feng et al., 2019). Pharmacological blockade of NLRP3 (Zhang et al., 2015) or its genetic deletion (Su et al., 2017) decreased the level of inflammatory mediators and counteracted the symptoms of depressive-like behavior. Microglia has been shown to be essential for these effects, because chronic minocycline treatment known to block the activation of microglia inhibited the following engagement of the NLRP3 inflammasome and the ensuing increased release of inflammatory mediators (Wang et al., 2018). Further, the inflammasome inhibitor Ac-Tyr-Val-Ala-Asp-chloromethyl ketone blocked the behavioral alterations and the production of inflammatory mediators caused by systemic injection of LPS to mice (Zhu et al., 2017). Chronic treatment with the standard antidepressant drug fluoxetine suppressed all symptoms induced by UCMS in rodents (Pan et al., 2014; Du et al., 2016). There is a multitude of review articles available which give further insight to the causal relationship between inflammasome activation and MD (Alcocer-Gómez et al., 2016; Kaufmann et al., 2017; Franklin et al., 2018; Herman and Pasinetti, 2018).

The role of P2X7Rs as essential activators of NLRP3 was also convincingly demonstrated by showing that UCMS elevates hippocampal P2X7R levels (Tan et al., 2017). The selective P2X7R antagonist Brilliant Blue G (BBG) attenuated the increase of immobility time in TST and FST in mice after activation of the inflammasome by LPS (Ma et al., 2014). Similarly, BBG reversed the behavioral deterioration induced by UCMS in mice (Farooq et al., 2018), and clemastine, a nonselective antagonist of P2X7Rs, counteracted the prolonged duration of immobility in TST (Su et al., 2018).

Another piece of evidence for the participation of P2X7Rs in depressive-like behavior was supplied by the use of KO animals. *P2RX7*^−/−^ mice exhibited an antidepressant-like profile in TST and FST; this effect was not accompanied by changes in spontaneous locomotor activity (Basso et al., 2009). In these animals, decreased behavioral despair in FST, reduced immobility in TST, and attenuated amphetamine-induced hyperactivity were detected, indicating an antidepressant phenotype (Sperlágh et al., 2012; Csölle et al., 2013a,b). In addition, several potential mechanisms were identified for these mice such as elevated basal production of brain-derived neurotrophic factor (BDNF), enhanced neurogenesis, and increased 5-HT bioavailability in the hippocampus (Csölle et al., 2013b). In contrast to these findings, equivalent levels of immobility were observed in *P2RX7*^−/−^ and WT mice on the first exposure to forced swim, but much greater immobility was seen in the WT animals on second and third exposures (Boucher et al., 2011). An explanation for this discrepancy may be that the FST was recently questioned to be an adequate model of despair or helplessness (Molendijk and de Kloet, 2019).

Another factor of insecurity inherent to the P2X7R-deficient mice is that with the two types used routinely for experimentation, some splice variants of the *P2RX7* gene escape inactivation (Bartlett et al., 2014; Sperlágh and Illes, 2014). Experiments with a recently generated conditional humanized P2X7R-deficient mouse, supposed to be devoid of active splice variants of the receptor, could be helpful in this respect (Metzger et al., 2017b).

A further argument for the involvement of P2X7Rs in the etiology of MD is supplied by studies which show that inhibition or genetic abrasion of the P2X7R–Panx-1 pore complex suppresses spreading depolarization and neuroinflammation in mice (Chen et al., 2017). This is in perfect agreement with findings that Cx43- and Panx-1-based channels participate in the induction of neuroinflammation and cerebral neuropathies (Sarrouilhe et al., 2017; for further considerations on the role of connexins/pannexins in MD, see the section “Inhibited Astrocytic ATP Release in the Pre-frontal Cortex”).

## Microglial and Astroglial Functions; Cell Death and Proliferation by P2X7 Receptors

Microglia are the resident immunocytes of the CNS; unlike other tissue macrophages, they persist for the life of the organism with negligible turnover rates at steady state (Tay et al., 2016; Anderson and Vetter, 2019). Microglia are instrumental in the maintenance of biochemical homeostasis, neuronal circuit maturation during development, and experience-dependent remodeling of neuronal circuits in the adult brain (Szepesi et al., 2018; Anderson and Vetter, 2019; Illes et al., 2019). The cellular processes of quiescent or “resting” microglia are highly mobile (extension and withdrawal) by scanning the environment for disruptions of brain homeostasis (Davalos et al., 2005). When microglia detect danger signals, they rapidly become activated by shortening their processes, eventually being transformed to amoeboid microglia, which produces a number of cytokines, chemokines, and growth factors, as well as developing phagocytotic activity (Kettenmann et al., 2011).

Microglia establish close contact with both neurons (Eyo and Wu, 2013) and astrocytes (Jha et al., 2019), supplementing the “tripartite synapse” (see below) with a microglial component (“quadripartite synapse”; Schafer et al., 2013; Illes et al., 2019). An important regulator of this interaction is ATP/ADP, which is released from neurons and astrocytes/microglia by exocytotic and non-exocytotic mechanisms (Calovi et al., 2019). Microglia possess a range of P2Y receptors (P2YRs). P2Y1 receptors (P2Y1Rs) steer microglial migration (De Simone et al., 2010), P2Y6 receptors (P2Y6Rs) regulate microglial phagocytosis (Koizumi et al., 2007), and P2Y12 receptors (P2Y12Rs) are responsible for chemoattraction of microglial branches to the site of ATP accumulation (Ohsawa et al., 2010).

P2X7, the archetypical macrophage/microglial receptor, mediates two diametrically opposite functions of microglia such as, firstly, proliferation, most likely *via* calcium signaling (Monif et al., 2010, 2016), and, secondly, necrosis/apoptosis *via* the generation of transmembrane pores and activation of the caspase enzymatic cascade (Bartlett et al., 2014; He et al., 2017). Whereas microglial phagocytosis of bacteria and cellular debris is under the regulation of ATP/ADP, P2X7 has been shown to be a scavenger receptor for apoptotic cells even in the absence of its ligand ATP (Gu et al., 2011).

Astrocytes to a large extent define synaptic connectivity. Indirect effects are exerted by changes in astrocytic functions due to modifications in K^+^ uptake and redistribution; Cl^−^ and water fluxes; Na^+^/K^+^, Na^+^/Ca^2+^, or Na^+^/HCO_3_^−^ exchange; neurotransmitter uptake; etc (Verkhratsky et al., 2017; Mederos et al., 2018; Illes et al., 2019). Astrocytes also directly modify synaptic transmission, because they contact and partially ensheathe synapses with their perisynaptic processes (Allen and Eroglu, 2017). Astrocytes may release “gliotransmitters” [e.g., glutamate, γ-aminobutyric acid (GABA), and ATP] by an exocytotic mechanism modulating neuronal functions; the structural basis for this effect is the “tripartite synapse,” which consists of the presynaptic elements, the postsynaptic/dendritic structures, and the astrocytic processes terminating at the synapse (Araque et al., 1999; Halassa and Haydon, 2010; Illes et al., 2019). More recently, the tripartite synapse hypothesis has evolved into the idea of an “astroglial cradle” summarizing all aspects of the synapse function not only those mediated by neurotransmitters (Verkhratsky and Nedergaard, 2018). In addition, astrocytes may also deliver ATP/ADP into the extracellular space by non-exocytotic mechanisms *via*, for example, connexin hemichannels, pannexin channels, maxi-anion channels, and volume-regulated anion channels, contributing to the exocytotic release (Cheung et al., 2014; Dahl, 2015).

For a couple of years, it was doubted whether astrocytes possess the prototypic microglial P2X7R (see, e.g., Jabs et al., 2007); however, more recently convincing functional evidence corroborated this notion (Duan et al., 2003; Oliveira et al., 2011; Illes et al., 2012, 2017). Immunohistochemical investigations in the nucleus accumbens of rats showed that after stab wound injury, P2X7R immunoreactivity was observed in glial fibrillary acidic protein (GFAP)-positive astrocytes (Franke et al., 2001). Similar findings were reported for the cerebral cortex of spontaneously hypertensive rats, where the occlusion of the medial cerebral artery led to the upregulation of P2X7Rs in the penumbra surrounding the necrotic region (Franke et al., 2004). Thus, it was concluded that P2X7Rs induce proliferation of astrocytes upon their stimulation by ATP possibly released from the nearby, massively damaged CNS tissue (Franke et al., 2012; Franke and Illes, 2014; Martin et al., 2019).

## Inhibited Astrocytic ATP Release in the Prefrontal Cortex and Hippocampus Is a Possible Link to Mood Disorders

After having discussed the role of P2X7Rs in MD and BD, we turn our attention to a possible role of astrocytes in the pathogenesis of mood disorders by their impeded release of ATP. Numerous lines of evidence support the contention that modification of astrocytic functions or decreased density of astrocytes in the frontolimbic and hippocampal regions is associated with depression (Rajkowska and Stockmeier, 2013; Peng et al., 2015; Rial et al., 2016). Astrocytes are integrated into networks where individual cells communicate with each other *via* gap junctions. Connexins, mainly represented by Cx43, provide the molecular basis for gap junction channels, connecting the cytoplasm of adjacent glial cells (Theis and Giaume, 2012; Verkhratsky and Nedergaard, 2018; Illes et al., 2019). These channels allow direct exchange of a variety of small molecules of less than about 1 kDa, including ions (most importantly Ca^2+^), energy metabolites, neurotransmitters, and signaling molecules coordinating metabolic and functional activities of connected cells (Pannasch and Rouach, 2013; Cheung et al., 2014). In addition, unopposed connexin hemichannels and Panx-1 channels are conduits for ATP release from astrocytes (Huang et al., 2012; Beckel et al., 2014).

Rats exposed to chronic unpredictable stress exhibited deficits in the sucrose preference test, which signals anhedonic behavior, a core symptom of depression. In the prefrontal cortex of these animals, the diffusion of gap junction channel-permeable dyes as well as the expression of Cx43 is decreased (Sun et al., 2010; Xia et al., 2018). The infusion into the prefrontal cortex of both the gap junction blocker carbenoxolone and the Cx43 mimetic, antagonistic peptide Gap27 caused anhedonia. Similarly, exposure to chronic unpredictable stress of rats also caused a decrease in the expression of prefrontal cortical connexins, while long-lasting treatment with antidepressants with unrelated structure and mode of action invariably increased the expression of connexins (Ren et al., 2018).

In the case of the blockade of connexins, it is unclear whether the gap junction property or the outflow of various neuroactive substances, for example, ATP through (hemi)channels, has been inhibited in the above experiments. However, Panx-1 works only as a channel, and therefore, its blockade in the medial prefrontal cortex by carbenoxolone, ^40^Panx, and mefloquine appeared to be due to impaired release of an astrocytic signaling molecule (Ni et al., 2018). This molecule may be ATP, because the mefloquine-induced depressive-like behavior was prevented by preconditioning with ATP.

However, opposite results have also been published. Dye uptake experiments in hippocampal slices demonstrated that acute restraint stress, known to instigate depressive-like behavior, induced opening of both Cx43 and Panx-1 channels (Orellana et al., 2015). Moreover, incubation of cultured astrocytes with seven antidepressants inhibited Cx43 channels with different efficacies depending on their therapeutic potencies (Jeanson et al., 2015). An explanation for these divergent results may be that conclusions were drawn based on investigations carried out on different organizational structures (cell culture/brain slice vs. whole animal) and different areas of the brain (hippocampus vs. prefrontal cortex).

When mice susceptible or non-susceptible to social defeat were compared to each other, the brains of the susceptible mice contained lower ATP levels than those of the non-susceptible ones (Cao et al., 2013). Further, FST also caused ATP deficiency in the brain and decreased the ATP content in the microdialysates of their prefrontal cortices. The infusion of ATP into the lateral ventricle of the mouse brain decreased the duration of immobility in the FST. Inositol 1,4,5-trisphosphate (IP3) triggers the release of Ca^2+^ from the endoplasmic reticulum which is a prerequisite for the exocytotic release of ATP. In consequence, IP3 receptor type 2 KO mice exhibited lower ATP release from astrocytes compared with their WT counterparts, as well as a depressive-like phenotype. Comparably, the astrocytic, vesicular release of ATP was blocked, when in mice, a dominant negative domain of vesicular soluble N-ethylmaleimide-sensitive fusion protein attachment protein receptor (SNARE) was selectively overexpressed in astrocytes. These transgenic animals also exhibited depressive-like behaviors (Halassa and Haydon, 2010).

Conventional KO and conditioned astrocytic KO of the calcium homeostasis modulator 2 channel (Calhm2) initiated depression-like behaviors in mice (TST and FST), indicating that this channel is the exit pathway for the release of ATP (Jun et al., 2018). In partial disagreement with these findings, the effect of the antidepressant drug fluoxetine has been shown to increase ATP exocytosis (Kinoshita et al., 2018). In consequence, the authors of this latter study concluded that the astrocytic release of ATP involved in depression operates by vesicular exocytosis rather than by Calhm2 opening.

Thus, ample evidence supports the notion that an impaired ATP release from prefronto-cortical astrocytes is the primary reason for depressive-like behavior and probably also MD. However, there is disagreement on whether this damage may be confined to connexin/pannexin hemichannels, the Ca^2+^-dependent exocytotic machinery, or Calhm2 channels as exit pathways for ATP. In view of the already discussed idea that hyperreactivity of microglial/astrocytic P2X7Rs is causally involved in the pathogenesis of MD/BD, it is quite attractive to hypothesize that the decreased ATP concentration in the prefrontal cortex (and hippocampus) leads to upregulation of P2X7Rs in this area of the brain.

## P2X7R Antagonists as Possible Therapeutic Agents to Treat Mood Disorders

Because P2X7Rs mediate peripheral and central inflammation, a number of pharmaceutical companies developed ligands for this target, and some of them advanced P2X7R antagonistic compounds even to clinical trials. A major advantage of a P2X7R antagonistic drug would be that the receptor is stimulated only by pathologically high extracellular concentrations of ATP; thus, its blockade will not interfere with effects due to the more physiological release of smaller quantities of ATP (Illes et al., 2019). Although, against expectations, P2X7R antagonists did not produce a beneficial effect on rheumatoid arthritis (Keystone et al., 2012; Stock et al., 2012), they improved symptoms in patients with moderate-to-severe Crohn’s disease (Eser et al., 2015). Nonetheless, the development of such compounds for both therapeutic indications was terminated by Pfizer and Astra-Zeneca (Rech et al., 2016; Young and Górecki, 2018), in the case of Crohn’s disease probably also because of insufficient safety margins (Bhattacharya and Biber, 2016).

It can be derived from the available literature as discussed in our review that P2X7Rs may be promising targets to treat MD and BD (Bhattacharya, 2018; Wei et al., 2018). However, the following three difficulties are major obstacles in developing new P2X7R antagonists for the treatment of mood disorders: (1) a number of P2X7R antagonists act in rodent receptor orthologs but not in human receptor orthologs when investigated under *in vitro* conditions (Bhattacharya and Biber, 2016); (2) the disease can be modeled by depressive-like states induced by applying acute or chronic inescapable stress to rodents; however, it is most likely that there is no perfect analogy with the human disease (Ribeiro et al., 2019); and (3) P2X7R antagonists have to pass the blood-brain barrier in order to exert effects in the CNS.

The majority of compounds disclosed in the last decade are human-specific P2X7R antagonists with no or weak rodent activity but suffer from lack of robust CNS permeability. However, numerous blood-brain barrier-permeable P2X7R antagonists have been developed by Abbott, Astra-Zeneca, GlaxoSmithKline, and especially Janssen more recently (Bhattacharya, 2018; Wei et al., 2018). The Janssen compounds JNJ-47965567 (Bhattacharya et al., 2013) and JNJ-42253432 (Lord et al., 2014) demonstrated activity in rodent and human P2X7Rs, had good rat pharmacokinetic profiles, and had excellent brain penetration, when dosed subcutaneously.

## Conclusions and Perspectives

A tight causal relationship of P2X7Rs with mood disorders is imperatively suggested by their involvement in neuroinflammation and the subsequent modulation/damage of neuronal circuits in mood-relevant areas of the brain. Functional changes in long-term synaptic potentiation (LTP) in the lateral habenula have been observed in rats exposed to inescapable stressors leading to learned helplessness (Li et al., 2011; Park et al., 2017). Similarly, in models of learned helplessness, the expression of synapse-related genes decreased, indicating the loss of synaptic structures. The morphological alterations were manifest as a decrease in spine synapse density in the CA1, CA3, and dentate gyrus of the hippocampus (Hajszan et al., 2009) and were absent in P2X7R-deficient mice (Otrokócsi et al., 2017).

In short, neuroinflammation triggered by inescapable stressors activates microglial cells outpouring cytokines/chemokines, proteases, reactive oxygen, and nitrogen species which damage neurons in the prefrontal cortex and hippocampus (Box 1). Microglia also acquires phagocytotic properties, thereby shaping adult neuronal circuits by phagocytosis and synaptic stripping. The classical DAMP ATP initiates the transformation of ramified microglia to microglia with a rounded surface. P2X7Rs have a key role in microglial activation causing *via* multiple signal transduction pathways functional/morphological changes leading to depressive-like reactions in animals and possible also in humans.

Box 1 **Microglial cellular effectors modulating neuronal functions**

- Resting (ramified) microglia constantly scan their environment for exogenous and endogenous signals indicating a threat to the neuronal homeostasis. They detect PAMPs such as LPS from bacterial infection or DAMPs, such as ATP. DAMPs initiate the transformation of ramified microglia after withdrawal of their cellular processes to microglia with a rounded surface.
- In activated microglia, the assembly/activation of the inflammasome converts pro-caspase-1 to caspase-1, which in turn cleaves the biologically inactive pro-IL-1β to IL-1β. Caspase-1 also activates the apoptotic caspase enzyme cascade to induce programmed cell death (apoptosis). After LPS priming, P2X7Rs largely boost the inflammatory cytokine response executed in the first line by IL-1β, but also by IL-6 and TNF-α.
- Microglial P2X7Rs are termed “suicide receptors” because their activation causes necrosis/cell death.
- Activated microglia release proteases as well as reactive oxygen and nitrogen species into their cellular environment. In addition, they secrete diacylglycerol lipase responsible for endocannabinoid production. These microglia also release ATP and probably also glutamate by vesicular exocytotic mechanisms.
- ATP through activation of microglial P2X7Rs releases extracellular vesicles from the plasma membrane (microvesicles and exosomes), inducing a robust inflammatory reaction in glial cells.
- Activated microglia also acquires phagocytotic properties, thereby eliminating not only cellular debris or pathogenic bacteria but also surplus neurons during development and thereby shaping adult neuronal circuits by phagocytosis or synaptic stripping.

In view of good blood-brain barrier-permeable P2X7R antagonists at our disposal and the intensive research activities carried out in academic and pharmaceutical institutions, there is strong hope that newly synthesized and clinically tested drugs of this family will be soon available as potent and side-effect-free antidepressants.

## Author Contributions

PI drafted the manuscript. All authors contributed to manuscript revision and read and approved the submitted version.

## Conflict of Interest

The authors declare that the research was conducted in the absence of any commercial or financial relationships that could be construed as a potential conflict of interest.
